# Salinity alleviator bacteria in rice (*Oryza sativa* L.), their colonization efficacy, and synergism with melatonin

**DOI:** 10.3389/fpls.2022.1060287

**Published:** 2023-01-12

**Authors:** Amrita Gupta, Rajesh Kumar Tiwari, Renu Shukla, Arvind Nath Singh, Pramod Kumar Sahu

**Affiliations:** ^1^ Amity Institute of Biotechnology, Amity University Uttar Pradesh, Lucknow, India; ^2^ Indian Council of Agricultural Research (ICAR)-National Bureau of Agriculturally Important Microorganisms, Kushmaur, Maunath Bhanjan, India; ^3^ Indian Council of Agricultural Research (ICAR)-Indian Institute of Seed Sciences, Kushmaur, Maunath Bhanjan, India

**Keywords:** rhizospheric bacteria, endophytes, salt tolerance, PGP traits, colonization under stress

## Abstract

In this study, rhizospheric and endophytic bacteria were tested for the alleviation of salinity stress in rice. Endophytic isolates were taken from previous studies based on their salt stress-alleviating traits. The rhizospheric bacteria were isolated from rice and screened based on salt tolerance and plant growth-promoting traits. Molecular identification indicated the presence of class Gammaproteobacteria, Bacillota, and Actinomycetia. Two-two most potential isolates each from rhizospheric and endophytic bacteria were selected for *in planta* trials. Results showed that microbial inoculation significantly improved germination and seedling vigor under elevated salinity. The confocal scanning laser microscopy showed higher bacterial colonization in inoculated rice roots than in control. Based on this experiment, rhizospheric bacteria *Brevibacterium frigoritolerans* W19 and endophytic *Bacillus safensis* BTL5 were selected for pot trial along with a growth-inducing compound melatonin 20 ppm. Inoculation of these two bacteria improved the levels of chlorophyll, proline, phenylalanine ammonia-lyase, catalase, superoxide dismutase, polyphenol oxidase, root-shoot length, and dry weight under elevated salt concentration. The gene expression studies showed modulation of *SOD1, CATa, NHX1*, and *PAL1* genes by the bacterial strains and melatonin application. The inoculation was found to have additive effects with 20 ppm melatonin. This enhancement in dry matter accumulation, compatible solute production, and oxidative stress regulation could help plants in mitigating the ill effects of high salinity. Exploring such a combination of microbes and inducer molecules could be potentially useful in developing stress-alleviating bioformulations.

## 1 Introduction

Rice (*Oryza sativa* L.) is a staple crop offering food to >2 billion human population (Ziska et al., 2015) and the significance further intensifies with the projected population growth, expected to reach 9.7 billion by 2050 (World Population Prospects, 2019). On the other hand, the acreage of arable soil is diminishing, and increasing soil salinity is one of the major causes behind it, affecting >20% of irrigated land globally (Shrivastava and Kumar, 2015). Soil salinity negatively alters plant growth, production, and grain quality. One of the deleterious effects is salt-induced germination failure and growth retardation in seedlings (Hakim et al., 2010; Bai et al., 2017). Despite the huge commercial importance of different Basmati rice varieties, soil salinity affects germination, early seedling growth, ion content, and other physiological parameters (Jamil et al., 2012).

Various plant growth-promoting bacteria (PGPB) have the potential to alleviate the deleterious effects of salinity (Shahzad et al., 2017; Kashyap et al., 2019; Bakhshandeh et al., 2020). Khan et al. (2020) showed that halophytic endophytes from genera *Curtobacterium, Enterobacter, Bacillus*, and *Micrococcus* resulted in enhanced growth of Waito‐C rice under salinity stress. It is necessary to explore ways to improve the growth and performance of the seedlings at the nursery level, as the cost of seeds contributes to a major portion of the cost of cultivation. Early growth improvement has greater significance in the good plant stand. Thus, microbes that could enhance seedling establishment could directly contribute to better yield. Different phytohormones produced by the microbes are reported to improve seed germination and growth (Shahzad et al., 2017; Khan et al., 2020).

Microbes having the potential to enhance the availability of nutrients in the rhizosphere could compensate for the nutrient deprivation caused by high salinity (Jiménez-Mejía et al., 2022). The exopolysaccharides (EPS) production and the formation of biofilms that accumulate EPS around the rhizosphere reduce the available Na+ content around the plant roots, thus reducing the harmful effects of soil salinity (Singh and Jha, 2016; Sagar et al., 2022). Also, enhancement of nutrient uptake by phosphate solubilizing microorganisms, zinc solubilizing microorganisms, iron quenchers, phytohormone producers/inducers, etc. could help plants grow under saline conditions (Sahu et al., 2021a; Jiménez-Mejía et al., 2022). Therefore, microbes having the potential for production of exopolysaccharides, siderophores, IAA, and solubilization of plant nutrients like phosphorus, and Zn, could guard the plants against salinity stress to some extent and supplement nutrition to keep the plants healthy. Since microbial diversity is huge with varied functional behavior, exploring a wide diversity of stress-tolerant microorganisms could be a viable option to help overcome the germination problem in saline areas. Thus, in the present study, both endophytic and rhizospheric microbes were screened to harness benefits from both niches.

Apart from the plant growth promoting potential of the microorganisms, colonization efficiency is a major factor that affects their function. Effective colonization in the plant system indicates inoculum efficacy for producing plant health and growth promotion activities, referred to as rhizosphere competence (Raaijmakers and Weller, 2001) in the case of rhizospheric microbes, and as internal colonization competence for an endophyte realizing its functional potential (Compant et al., 2005). Colonization and its further survival in the rhizosphere and endosphere are also important to evaluate the effectiveness of delivery techniques (Compant et al., 2005). Although several reports suggest the evaluation of colonization competence of applied inoculants by different techniques such as molecular tracking (Ownley et al., 2008), electron microscopy (Ownley et al., 2008), *gfp* and *gus*A (Compant et al., 2005), qPCR (Barelli et al., 2018), confocal scanning laser microscopy (Álvarez et al., 2020; Sahu et al., 2020), etc., the colonization with respect to the presence of elevated stress is yet to be explored in greater detail. So, this study was done to find out how well endophytic and rhizospheric microbes colonize plants when they are under salt stress.

In addition to the microbial potential, several plant growth-inducing compounds have been screened for their effects in alleviating abiotic stresses. Melatonin (N-acety-5-methoxytryptamine) is one of them, which is being explored for growth induction and stress alleviation (Liang et al., 2017). It was found to induce root growth, photosynthetic pigments, reactive oxygen species scavengers, etc. and to reduce electrolyte leakage under elevated salinity (Altaf et al., 2021). Despite the potential roles of melatonin, the combined effects of melatonin with microbes are the least worked out. Therefore, in this study, the effects of potential microbes on salinity-induced germination failure, root colonization, and their complementation with melatonin were assessed. The present investigation was focused on exploring microbes from different niches for salinity alleviation, colonization competence under salt stress, and compatibility with melatonin to improve plant performance under salinity.

## 2 Material and methods

### 2.1 Microbial cultures and characterization for salinity alleviation traits

#### 2.1.1 Microbial cultures

Rhizosphere soil samples were isolated from six paddy fields from the saline-sodic patches of the Indo-Gangetic plains of Eastern Uttar Pradesh. Two bacterial endophytes, potential alleviators for biotic and abiotic stress tolerance, *Bacillus haynesii* 2P2 from *Solanum lycopersicum* (Sahu et al., 2019) and *Bacillus safensis* BTL5 from *Ocimum tenuiflorum* (Sahu et al., 2021b), were taken from PMI and RB Lab, ICAR-NBAIM and stored in glycerol stock till further use.

#### 2.1.2 Growth under hypersaline conditions and production of exopolysaccharides

The rhizospheric isolates were screened for salt tolerance. An actively growing bacterial suspension (24 h old) was spotted onto the nutrient agar medium enriched with five different NaCl concentrations (400, 800, 1200, 1600, and 2000 mM) and incubated for 24 h at 28 ± 0.5°C. Production of EPS was qualitatively assayed as per the method described in Siddikee et al. (2011). For 5 days, the rhizospheric isolates were grown in 50 mL minimal media in a shaker (150 rpm) at 28 ± 0.5°C. The cultures were centrifuged (10000g for 10 min) and the supernatants were collected. The formation of a precipitate after the addition of ice-cold ethanol indicated a positive reaction for EPS production.

### 2.2 Characterization for growth-promoting traits *in-vitro*

The phosphate (P) solubilization capacity of rhizospheric isolates was determined by spotting actively grown cultures in modified Sperbers’ agar medium as described in Sahu (2012). After incubation for 48 h, the development of a clear halo around the spots was considered a positive response for P-solubilization. Siderophore producing capacity was determined as per the methodology of Schwyn and Neilands (1987), by the chrome azurol S assay. The formation of the orange halo zone around the colonies after 48 hours of incubation at 28 ± 0.5°C indicated a positive response for siderophore production. Zinc (Zn) solubilization by bacterial strains was assessed as per the method described in Fasim et al. (2002), following incubation for 48 h at 28 ± 0.5°C in mineral salts agar medium supplemented with 0.1% of insoluble ZnO. Solubilization of insoluble zinc compound resulted in a clear zone formation around the spots. Production of ammonia by the bacterial isolates was assessed using the method described in Cappuccino and Sherman (1992) with slight modifications. In brief, 24 hour old (diluted to 0.2 OD) cultures were inoculated into 5 mL of peptone broth and incubated for 72 hours at 28 ± 0.5°C. After incubation, each culture was centrifuged and the supernatant was mixed with 0.5 mL of Nessler’s reagent. The development of yellow-brown to dark brown was considered a positive response for ammonia production. Indole-3-acetic acid (IAA) production by bacterial isolates was studied as per the method described elsewhere (Patten and Glick, 1996). The intensity of the developed pink color was read at 530 nm for quantitative measurement.

### 2.3 Molecular identification of bacterial isolates

**Figure 1:**
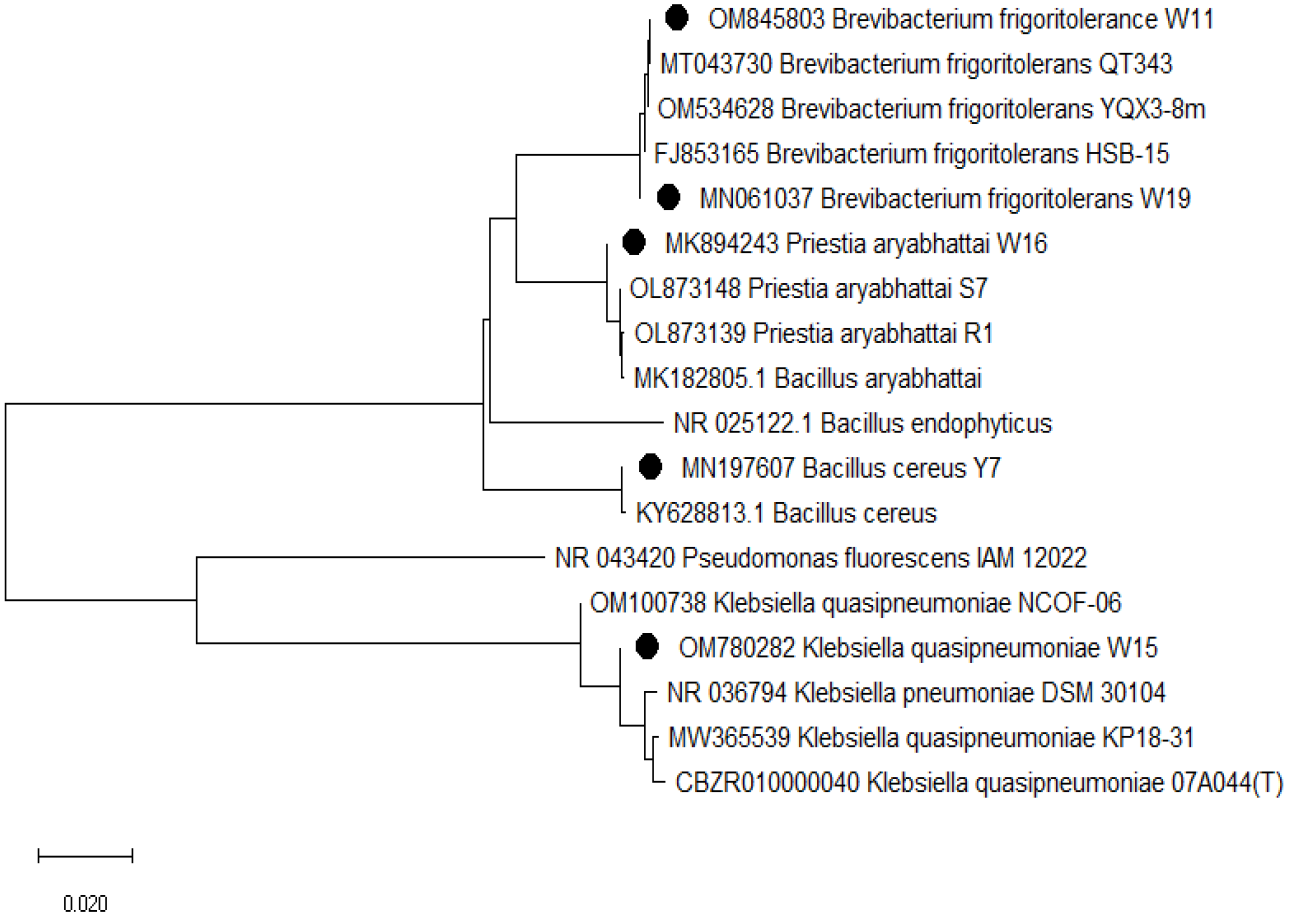
Phylogenetic tree indicating the evolutionary relationship of the bacterial isolates under study with other close relatives as inferred by the Neighbor-Joining (NJ) method in the program MEGA 7 (version=7.0.25). Bootstrap analysis was undertaken on 1000 random samples assessed from the multiple alignments.

Freshly grown cultures (24 h old) were used for isolating DNA from rhizospheric isolates using the Genetix Nucleopore kit following the manufacturer’s instructions. PCR amplification of the 16S rRNA gene was done using primers PA forward (5’-AGAGTTTGATCCTGGCTCAG-3’) and PH reverse (5’-AAGGAGGTGATCCAGCCGCA-3’) with a target product length of 1500 bp (approx). The amplified 16S rDNA was purified and sequencing was done by the Sanger method. The sequences obtained were curated and the sequence similarity was checked in the EZbiocloud database (https://www.ezbiocloud.net) as well as with the NCBI Blastn program. Sequences with the nearest match identities were submitted to the NCBI-GeneBank database. The phylogenetic tree was constructed using the neighbor-joining method in MEGA7 (version=7.0.25) (**Figure 1**).

### 2.4 *In-vivo* experiments

Based on salinity tolerance and plant growth promoting traits, two rhizospheric isolates (W15 and W19) along with two endophytic isolates (2P2 and BTL5) were selected for *in vivo* studies. In the first experiment, the effects on germination, seedling emergence, and colonization potential were tested on the rice cultivar Pusa Basmati-1 (PB-1) in a roll towel assay. In the second trial, one rhizospheric (W19) and one endophytic strain (BTL5) were used in a pot trial with a growth inducer compound, melatonin.

#### 2.4.1 Effect on salinity-induced germination failure and seedling vigor

Surface sterilized rice seeds were inoculated with 24-hour cultures and the mixtures incubated for 30 min. Three sets of treatments were made, one without additional salt, one with 100 mM NaCl, and one with 200 mM NaCl. The treatments in each set were (T1) Control, (T2) W15, (T3) W19, (T4) 2P2, and (T5) BTL5. Twenty-five seeds were placed on sterile germination paper and rolled. The assembly is placed in sterile conditions and incubated for 2 weeks.

#### 2.4.2 Colonization under different salt concentrations

To understand the colonization behavior under elevated salinity, root samples from two salt concentrations (i.e. control and 200 mM NaCl) from roll towel assay were taken for the colonization experiment. The fine roots were stained with the LIVE/DEAD™ *Bac*Light™ bacterial viability kit (Invitrogen, USA) as described elsewhere (Sahu et al., 2019) and visualized using a Confocal Scanning Laser Microscope (CSLM; Nikon Eclipse 90i) under a 488 nm channel. The artifacts arising from autofluorescence were avoided by comparing the samples with un-inoculated control samples so that the fluorescence from bacterial colonization could be differentiated.

#### 2.4.3 Effect on salinity alleviation in pot trials

Two bacterial isolates from the first *in vivo* trial were used in pot trials with seven treatments and 3 replications each, in a randomized complete block design. The treatments were -T1= Negative control, T2= Positive control (200 mM NaCl), T3 = 200 mM NaCl + Melatonin 20 ppm, T4 = 200 mM NaCl + *Bacillus safensis* BTL5, T5 = 200 mM NaCl + *Brevibacterium frigoritolerans* W19, T6 = 200 mM NaCl + *Bacillus safensis* BTL5 + Melatonin 20 ppm, T7 = 200 mM NaCl + *Brevibacterium frigoritolerans* W19 + Melatonin 20 ppm. Pots were prepared and seedling inoculation was done as per the method described elsewhere (Sahu et al., 2019). Two paddy seedlings per hill having equal height, and two hills per pot were transplanted in pots. The salinity was maintained by 200 mM NaCl in respective pots. In the case of melatonin treatment, 20 ppm solution was sprayed on the foliar parts on 30 days old plants.

##### 2.4.3.1 Chlorophyll content

Leaves were harvested 50 days after transplanting for assessing chlorophyll content. One gram of leaf tissue was crushed in 80% pre-chilled acetone and the volume was made up to 100 mL. The absorbance of the supernatant was recorded at 645, 663, and 645 nm using a UV-vis 1700 spectrophotometer, Shimadzu, Japan. The amount of chlorophyll (mg/g) in leaf tissue was calculated by the formula given in Sadashivam and Manickam (1996).

##### 2.4.3.2 Proline content

Proline content in different treatments was measured 50 days after transplanting (DAT) by crushing 0.5 g sample in 10 mL 3% aqueous sulphosalicylic acid followed by filtering with Whatman no. 2 filter paper. Two mL of glacial acetic and 2 mL acid ninhydrin were added to 2 mL of filtrate. The mixture was kept in a boiling water bath for a period of 1 h. The reaction was terminated by keeping the mixture in an ice bath. Further, toluene (4 mL) was mixed by stirring for 20-30 sec, and the mixture was kept at room temperature for toluene layer separation. The upper layer was taken, and the absorbance of the red color was measured at 520 nm as described in Sadashivam and Manickam (1996).

##### 2.4.3.3 PAL content

Phenylalanine ammonia-lyase (PAL) content in shoot tissues was measured by preparing an enzyme extract as per the protocol of Havir (1987). Further, the enzyme extract (0.2 mL) was mixed with borate buffer (0.5 mL) and water (1.3 mL). In this mixture, one mL of L-phenylalanine solution was added to this mixture to initiate the reaction, which was incubated at 32 ± 0.5°C for 60 min. The reaction was stopped by adding 0.5 mL of 1 M Tri-chloroacetic acid. The absorbance at 290 nm was measured using a UV-vis 1700 spectrophotometer.

##### 2.4.3.4 Antioxidant enzymes

The activities of catalase (CAT), superoxide dismutase (SOD), and polyphenol oxidase (PPO) in shoot tissues were assessed at 50 DAT following the protocol of Sadashivam and Manickam (1996). Briefly, 1 g of fresh plant tissue was ground in 3 mL of 0.1 M phosphate buffer (pH 7.0) and centrifuged at 12000 rpm for 15 min to prepare the enzyme extract. This enzyme extract was used for the estimation of CAT, SOD, and PPO. CAT activity was measured after mixing the enzyme extract (100 µl) with 3 mL phosphate buffer (50 mM). The reaction was started by adding 30 mL hydrogen peroxide (12.3 mM). The absorbance was recorded at 240 nm. In the case of SOD activity, the reaction mixture was prepared in 50 mM phosphate buffer (pH 7), and contained 1 mM EDTA, 13 mM methionine, 5 mM nitroblue tetrazolium and 300 µL of enzyme extract. In this reaction mixture, 30 µL of 2 mM riboflavin was added at the end and mixed well. The tubes were kept under light for 10 min to start the reaction, which was stopped by incubation in the dark. The absorbance was read at 560 nm. Polyphenol oxidase activity in rice leaves was determined by adding 100 µL of enzyme extract to a mixture containing 1.4 mL citrate phosphate buffer (0.1 M), 0.5 mL 1,3,5-Trinitrobenzene, and 1 mL catechol solution (2 mM). Absorbance was read after adding the enzyme extract at 412 nm in 30 sec intervals for 3 min.

##### 2.4.3.5 Gene expression study

The total plant RNA was isolated using PureLink™ RNA mini Kit (Invitrogen, USA) following manufacturer’s instruction. Total RNA was converted to cDNA using High Capacity RNA-to-cDNA kit (Thermo Fisher Scientific, USA) following manufacturer’s instructions. Yield of cDNA among different treatments of two cultivars was assessed using nanodrop and a final concentration of 50 ng/µl was used uniformly for assessing the differences in the transcript levels among the treatments. The cDNA was be used for RT-PCR studies with key genes for salinity stress tolerance.

The gene expression study using RT-qPCR (Bio-Rad, USA) was taken up for four salt stress responsive genes (**Supplementary Table 1**). For this study, Eva Green SYBR Green Supermix Kit (Bio-Rad, USA) was used. Final concentration of gene-specific primers was maintained at 10pmol/µl and internal controls were utilized to determine and normalize the transcript level of mRNA. In the reaction mixture of 10 µl, 2 µl cDNA (50 ng/µl), 1.5 µl each of forward and reverse primers (10pmol/µl), and 5 µl real-time master mix. The RT-qPCR cycles were- denaturation at 95°C for 2 min; 40 repeats at 95°C for 30 s; 60°C for 30 s; and 72°C for 30 s. The fold changes in gene expression was assessed using 2-ΔΔCT method (Livak and Schmittgen, 2001) using endogenous gene Actin.

##### 2.4.3.6 Plant growth parameters

Root-shoot length and dry weight were recorded from different replicas of each treatment at 75 DAT. The pot soil was washed carefully to harvest the entire root mass. Dry weight was taken by drying the plant samples in a hot air oven at 70 °C until samples gained a stable weight.

### 2.5 Statistical analysis

In these studies with rhizospheric and endophytic bacteria, the experiments were performed in a randomized complete block design. Paper towel assay was performed with 15 treatments and three replicas each. The pot experiment was performed with 7 treatments and three replications each. Data were analyzed by one-way ANOVA and the means were compared by DMRT at p ≤ 0.05. The principal component analysis (PCA) biplot was done for growth attributes and ROS scavenging systems of rice plants using Displayr software.

## 3 Results

### 3.1 Isolation of rhizospheric microbes and characterization for salinity alleviation traits

**Figure 2:**
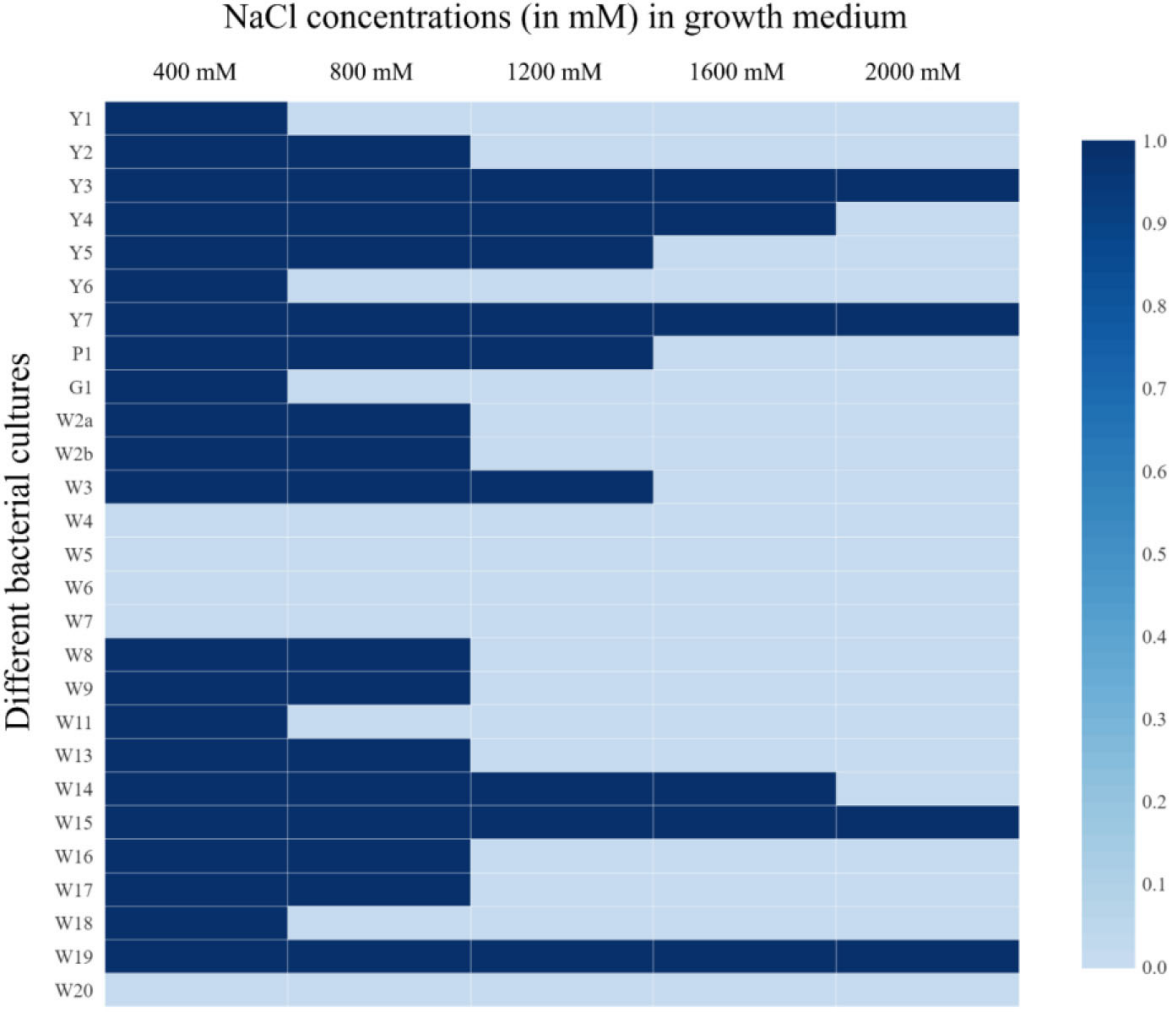
Salt tolerance of bacterial isolates from rice rhizosphere under 400, 800, 1200, 1600, and 2000 mM NaCl concentration; 1= Growth, and 0= No growth.

A total of 27 bacterial cultures were isolated from the rhizosphere soil of rice. These isolates were screened for growth under hypersaline conditions and shown to have differential tolerance to NaCl concentrations up to 2000 mM (**Figure 2**). More than 33% of the isolates could survive a salt concentration of 1200 mM. Amongst them, four isolates could survive even at a 2000 mM NaCl concentration. Exopolysaccharide production was also recorded from these plates. Among 27 isolates, 51.84% of isolates could produce EPS. In these, 14.81% of the isolates (Y1, P1, W15, and W16) were producing a good amount of EPS. Low EPS production was recorded from 37.03% of the isolates. In general, higher EPS production was recorded in the plates with relatively higher salt concentrations. Relatively higher EPS production was found in the plates amended with 1200 mM NaCl.

### 3.2 Characterization for growth-promoting traits *in-vitro*

**Figure 3:**
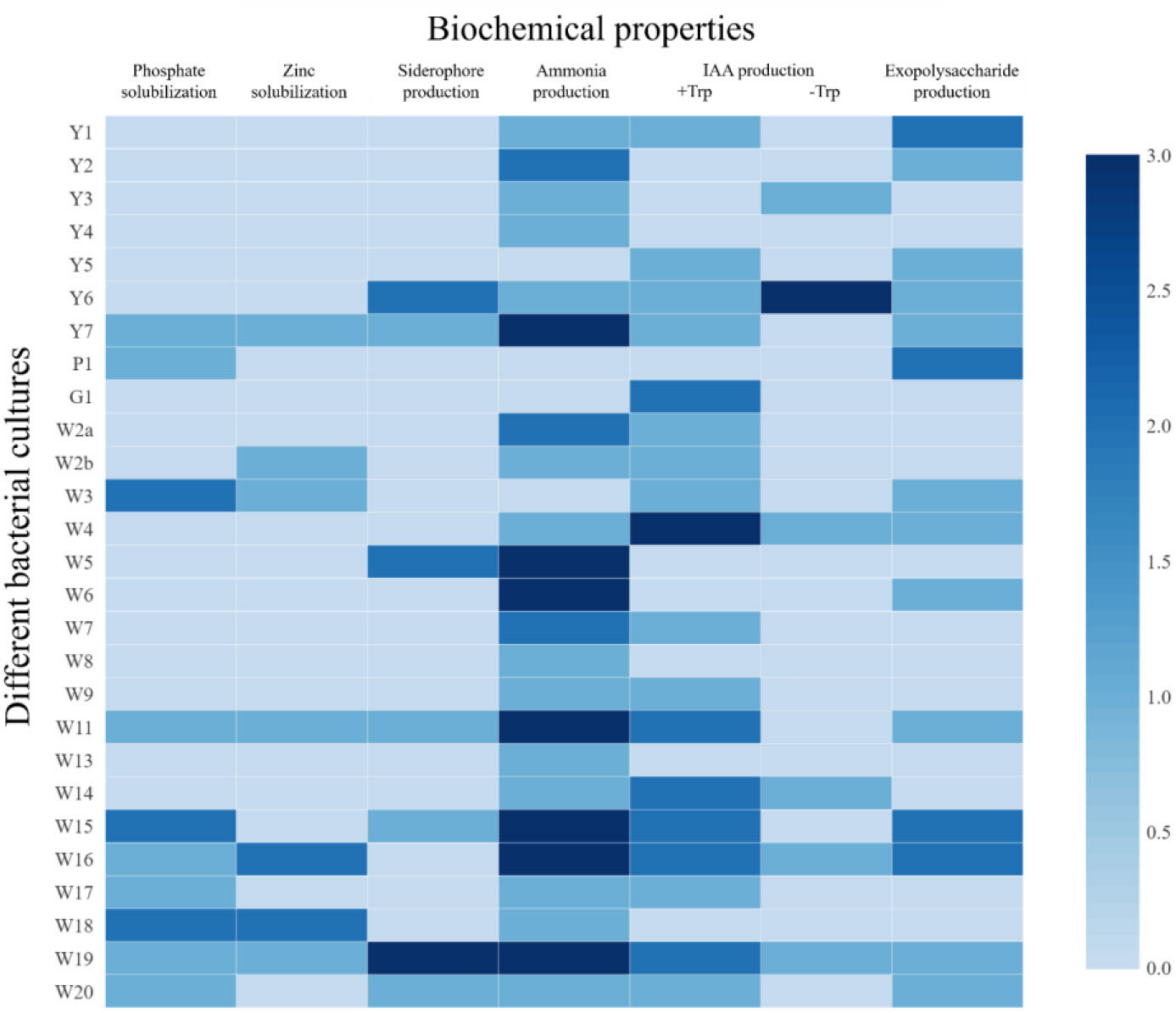
Different plant growth promoting attributes of the bacterial isolates from rice rhizosphere. For PSB, ZSB, and siderophore production: 0 = <10 mm, 1 = 10-12 mm, 2 = 12-14 mm, and 3 = > 14 mm (size of clear zone). For NH_3_ and EPS production: 0= no production, 1 = low, 2 = medium, and 3 = high. For IAA production (with and without Tryptophan): 0 = <5 µg/mL, 1 = 5-10 µg/mL, 2 = 10-15 µg/mL, and 3 = >15 µg/mL.

Among the rice rhizospheric isolates, ten isolates (37.03%) were found to be positive for P-solubilization (**Figure 3**). The largest zone size was reported from isolates W3, W15, and W18, followed by W17. In the case of Zn solubilization, results indicated that seven isolates were able to solubilize ZnO (**Figure 3**), and the largest zone was reported from W18 (12 mm). Siderophore production was found in 25.92% of the isolates. Results indicated that seven isolates were able to produce siderophores (**Figure 3**). The largest orange halo zone was reported for W19 (16 mm). Ammonia production was seen in 85% of the isolates. Among them, 26% of the isolates had higher ammonia production, whereas 11% and 48.10% of the isolates had medium and low ammonia production, respectively. In this qualitative measurement, the development of yellow to the pale yellow color indicated low ammonia production, the brown color indicated medium, and the development of dark brown color indicated higher ammonia production. In IAA production, both tryptophan-dependent and independent pathways were reported. Among tryptophan-dependent IAA producers, the highest production was reported for W4 (15.34 µg/mL) followed by G1 (12.22 µg/mL) and W14 (10.01 µg/mL). Among the tryptophan-independent IAA producers, Y6 was the highest IAA producer (15.78 µg/mL), followed by W16 (9.88 µg/mL), and W4 (9.71 µg/mL). However, tryptophan-dependent IAA production was more common (66.67%) than tryptophan-independent IAA production (22.22%).

### 3.3 Molecular identification

Based on 16S rRNA sequencing, the five isolates belong to classes Gammaproteobacteria, Bacillota (Firmicutes), and Actinomycetia (**Figure 1**). Isolates were from three genera (*Brevibacterium, Klebsiella*, and *Bacillus*) and four species (*Brevibacterium frigoritolerance* W11*, Klebsiella quasipneumoniae* W15*, Bacillus aryabhattai* W16*, Brevibacterium frigoritolerans* W19, and *Bacillus cereus* Y7). The 16S rRNA sequences of these isolates were deposited at NCBI (BankIt) with the accession numbers OM845803, OM780282, MK894243, MN197607, and MN061037.

### 3.4 *In-vivo* trials

#### 3.4.1 Effect on salinity-induced germination failure and seedling vigor

##### 3.4.1.1 Effect on germination

Among the non-saline treatments, there was no statistically significant difference (p ≤ 0.05) in germination percent. Under 100 mM NaCl, W19, 2P2, and BTL5 treatments resulted in significantly higher germination percentages as compared to the 100 mM Positive control and to W15. In the case of 200 mM NaCl treatments, all the bacterial inoculated treatments had significantly higher germination than the 200 mM NaCl control (**Table 1**).

**Table 1:** Seedling vigor parameters in different salt and microbe inoculation.

Sn.	Treatments	Germination percent	Mean root length (cm)	Mean shoot length (cm)	Mean root fresh wt. (g)	Mean shoot fresh wt. (g)	Vigor Index
1.	C	93.33 a	23.17 c	10.13 b	0.13 b	0.12 b	3112.67 b
2.	W15	100.00 a	25.42 bc	11.25 ab	0.16 ab	0.11 b	3666.67 ab
3.	W19	96.67 a	25.90 b	10.88 b	0.18 a	0.11 b	3551.17 ab
4.	2P2	100.00 a	27.03 ab	11.90 ab	0.15 ab	0.12 b	3893.00 a
5.	BTL5	96.67 a	28.70 a	12.80 a	0.17 a	0.14 a	4022.33 a
6.	C-100	76.67 b	13.33 c	5.58 b	0.11 c	0.05 b	1443.33 c
7.	W15-100	80.00 b	15.58 bc	7.37 a	0.15 a	0.08 a	1836.00 b
8.	W19-100	93.33 a	15.17 c	6.92 ab	0.15 a	0.10 a	2062.50 b
9.	2P2-100	93.33 a	18.22 ab	8.05 a	0.12 bc	0.09 a	2453.43 a
10.	BTL5-100	90.00 a	18.50 a	8.10 a	0.14 ab	0.09 a	2394.00 a
11.	C-200	53.33 b	5.00 b	1.12 b	0.09 b	0.03 d	329.83 b
12.	W15-200	73.33 a	5.58 b	1.25 b	0.12 a	0.04 c	498.33 b
13.	W19-200	76.67 a	8.17 a	2.82 a	0.13 a	0.05 b	844.00 a
14.	2P2-200	80.00 a	9.38 a	2.95 a	0.11 ab	0.05 b	986.67 a
15.	BTL5-200	83.33 a	9.20 a	3.02 a	0.12 a	0.06 a	1017.97 a

Means within columns of each of the three major rows with different letters are significantly different as determined by DMRT (p ≤ 0.05). The vigor index was calculated by multiplying germination percent by total plant length.

##### 3.4.1.2 Effect on plant height

Under the non-saline conditions, a significantly higher mean root length was recorded with the inoculation of BTL5 (28.70 cm), followed by 2P2 and W19, whereas the lowest was recorded in the control (23.17 cm), followed by W15 (**Table 1**). Under 100 mM NaCl, BTL5 and 2P2 treatments resulted in significantly higher root lengths, whereas under 200 mM NaCl, BTL5, 2P2, and W19 caused significantly higher mean root lengths. The mean shoot length was significantly higher with BTL5 (12.80 cm) followed by 2P2 and W15 inoculations, whereas the lowest was recorded in the control plants (10.13 cm). Under 100 mM NaCl, BTL5, 2P2, and W15 treatments led to significantly higher shoot length than the control, whereas, under 200mM mM NaCl, BTL5, 2P2, and W19 caused significantly higher mean shoot lengths.

##### 3.4.1.3 Effect on biomass accumulation

Under the non-saline conditions, a significantly higher mean root fresh weight was recorded with the inoculation of W19 (0.18 g) followed by BTL5 as compared to the control (**Table 1**). Under 100 mM NaCl, W15 and W19 treatments caused significantly higher (0.15g) root fresh weight, followed by BTL5, whereas under 200 mM NaCl, W19, W15, and BTL5 addition resulted in significantly higher mean root fresh weight. A significantly higher mean shoot fresh weight was recorded from the inoculation of BTL5 (0.14 g) as compared to all other non-saline treatments. Under 100 mM NaCl, all inoculated treatments yielded significantly higher shoot fresh weights than the control, whereas, under 200mM mM NaCl, BTL5 inoculation caused significantly higher mean shoot fresh weight (0.06 g) followed by 2P2, W19, and W15.

##### 3.4.1.4 Effect on seedling vigor

Among non-saline treatments, the seedling vigor index was highest in BTL5 (4022.33), and 2P2 (3893.00), followed by other treatments. Among the 100 mM NaCl treatments, 2P2 (2453.43) and BTL5 (2394.00) resulted in significantly higher vigor index. Under 200 mM NaCl, treatments with BTL5, 2P2, and W19 led to significantly higher vigor index (1017.97, 295 986.67, and 844.00, respectively) than the control and W15 treatments (**Table 1**).

#### 3.4.2 Colonization under different salt concentrations

**Figure 4:**
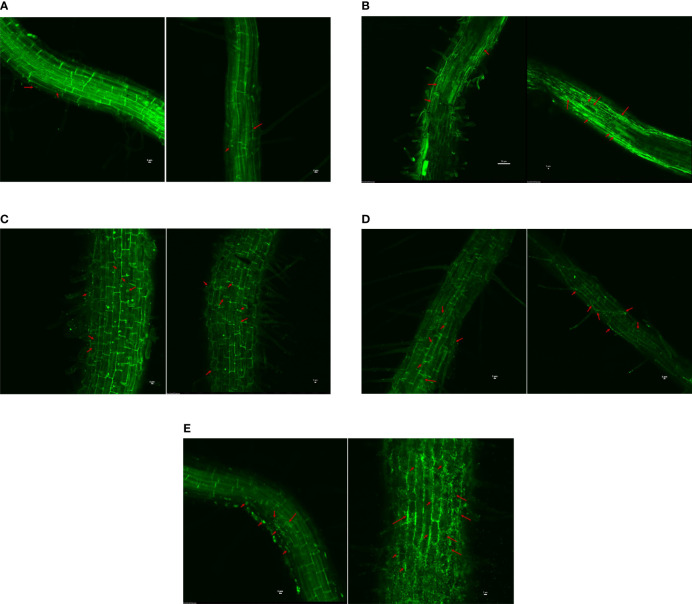
Confocal scanning laser microscopy to assess colonization efficiency of endophytes and rhizospheric bacteria at two salinity levels (with and without 200 mM NaCl), **(A)** Uninoculated Control, **(B)** *Klebsiella quasipneumoniae* W15, **(C)** *Brevibacterium frigoritolerans* W19, **(D)** *Bacillus haynesii* 2P2, and **(E)** *Bacillus safensis* BTL5. Red arrows indicate the colonization of bacteria in the plant roots of respective treatments.

The study indicated that the rhizospheric and endophytic isolates could colonize the rice root surface, at least to a concentration of 200 mM NaCl (**Figure 4**). There is a difference in colonization with an increase in salt concentration for the endophytes *B. safensis* BTL5 and *B. haynesii* 2P2. BTL5 treated plants were found to have good colonization, uniform in all the cells under salinity stress, whereas in 2P2 more signals were found in root hairs. In the case of both rhizospheric isolates (W15 and W19), colonization could be seen in both saline and non-saline treatments. The bacterial signals were relatively much less in the uninoculated control plants.

#### 3.4.3 Effect on salinity alleviation in pot trials

**Figure 5:**
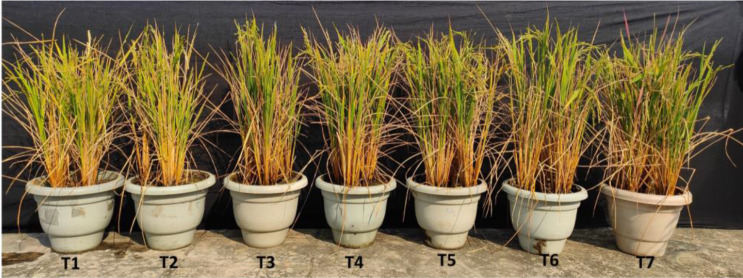
Effect of bacterial inoculation and melatonin in rice cultivar Pusa Basmati 1; T1=Negative control, T2=Positive control, T3 = 200 mM NaCl + Melatonin 20 ppm, T4 = 200 mM NaCl + *Bacillus safensis* BTL5, T5 = 200 mM NaCl + *Brevibacterium frigoritolerans* W19, T6 = 200 mM NaCl + *Bacillus safensis* BTL5 + Melatonin 20 ppm, and T7 = 200 mM NaCl + *Brevibacterium frigoritolerans* W19 + Melatonin 20 ppm. Error bars represent the standard deviation. The treatments with different letters are significantly different as determined by DMRT (p ≤ 0.05).

**Figure 6:**
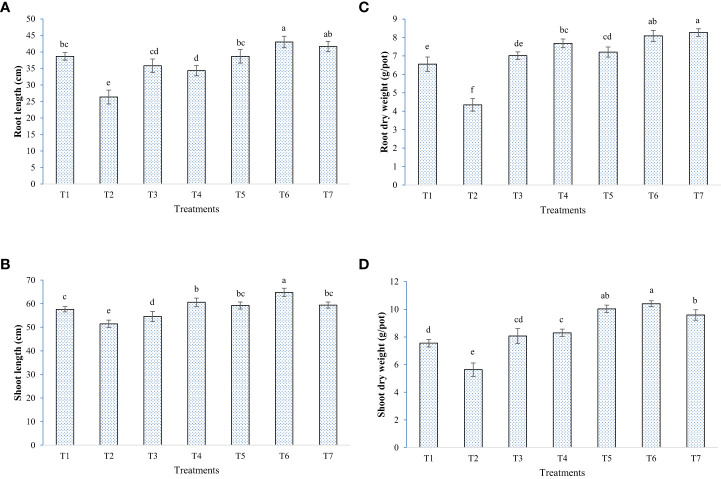
Effect of bacterial inoculation and melatonin in rice cultivar Pusa Basmati 1 (PB1), **(A)** Root length, **(B)** Shoot length, **(C)** Root dry weight, and **(D)** Shoot dry weight; T1=Negative control, T2=Positive control, T3 = 200 mM NaCl + Melatonin 20 ppm, T4 = 200 mM NaCl + *Bacillus safensis* BTL5, T5 = 200 mM NaCl + *Brevibacterium frigoritolerans* W19, T6 = 200 mM NaCl + *Bacillus safensis* BTL5 + Melatonin 20 ppm, and T7 = 200 mM NaCl + *Brevibacterium frigoritolerans* W19 + Melatonin 20 ppm. Error bars represent the standard deviation. The treatments with different letters are significantly different as determined by DMRT (p ≤ 0.05).

**Figure 7:**
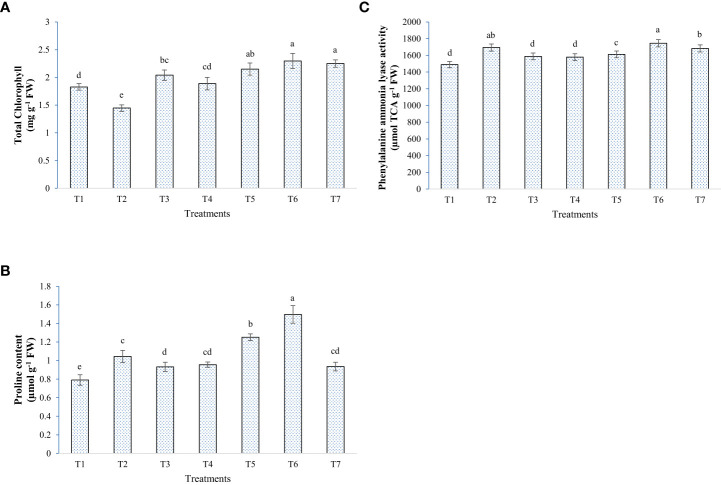
Effect of bacterial inoculation and melatonin in rice cultivar Pusa Basmati 1 (PB1), **(A)** Total chlorophyll content, **(B)** Proline content, and **(C)** Phenylalanine ammonia-lyase activity; T1=Negative control, T2=Positive control, T3 = 200 mM NaCl + Melatonin 20 ppm, T4 = 200 mM NaCl + *Bacillus safensis* BTL5, T5 = 200 mM NaCl + *Brevibacterium frigoritolerans* W19, T6 = 200 mM NaCl + *Bacillus safensis* BTL5 + Melatonin 20 ppm, and T7 = 200 mM NaCl + *Brevibacterium frigoritolerans* W19 + Melatonin 20 ppm. Error bars represent the standard deviation. The treatments with different letters are significantly different as determined by DMRT (p ≤ 0.05).

**Figure 8:**
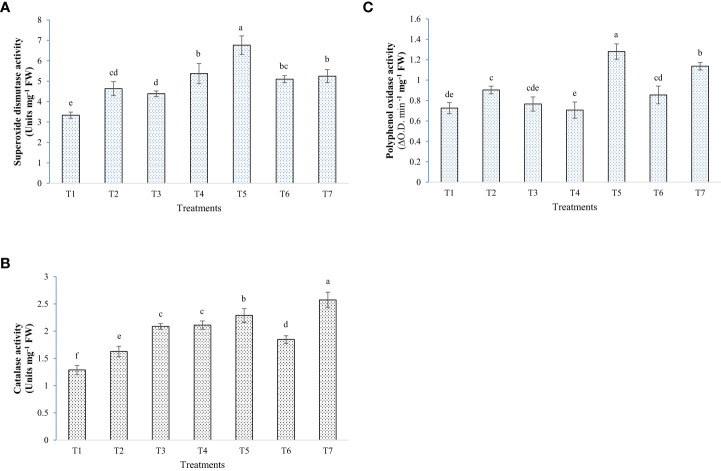
Effect of bacterial inoculation and melatonin in enzymatic antioxidants activities of rice, **(A)** Superoxide dismutase activity, **(B)** Catalase activity, and **(C)** Polyphenol oxidase activity; T1=Negative control, T2=Positive control, T3 = 200 mM NaCl + Melatonin 20 ppm, T4 = 200 mM NaCl + *Bacillus safensis* BTL5, T5 = 200 mM NaCl + *Brevibacterium frigoritolerans* W19, T6 = 200 mM NaCl + *Bacillus safensis* BTL5 + Melatonin 20 ppm, and T7 = 200 mM NaCl + *Brevibacterium frigoritolerans* W19 + Melatonin 20 ppm. Error bars represent the standard deviation. The treatments with different letters are significantly different as determined by DMRT (p ≤ 0.05).

In general, elevated salinity retarded the growth parameters (chlorophyll, plant height, and biomass accumulation) and enhanced the reactive oxygen species levels in the plant, as seen from enzymatic and non-enzymatic antioxidant activity (**Figures 5**–**8**). Inoculation of both bacterial strains could improve the root-shoot dry weight and reduce oxidative stress. Additionally, the spray of melatonin with inoculants has further enhanced the growth and development in a synergistic manner (**Figure 5**). The highest root dry weight was recorded for W19+melatonin treatment (8.27 g/pot) and BTL5+melatonin treatment (8.09 g/pot). In the case of shoot dry weight, BTL5+melatonin (10.41 g/pot) and W19 (10.03 g/pot) treatments led to significantly higher values than the other treatments. Application of melatonin increased biomass accumulation, however, inoculation of both BTL5 and W19 resulted in higher biomass accumulation than the melatonin alone. The reduction in growth was clearly visible for the Positive control treatment. Inoculation with microbes and melatonin improved chlorophyll content (**Figure 7**). Considering total chlorophyll, T5, T6, and T7 led to significantly higher content than the other treatments. Among the microbes applied, W19 could perform better than BTL5. However, plants with BTL5 + melatonin gave better synergistic effects on chlorophyll content than other treatments. The effect could also be seen for shoot length, which was the highest in plants treated with BTL5 + melatonin (64.78 cm) as compared to all other treatments. The plant height was affected by elevated salt, and the microbial and melatonin application helped plants to recover from salinity stress.

The application of microbes and melatonin had a mixed effect on the levels of the antioxidant systems of the rice plants under elevated salinity. Some of the treatments resulted in higher ROS scavenging activity than the Positive control, in opposition to some other treatments. PAL, CAT, and proline content were higher in the plants treated with microbes + melatonin, whereas in the case of SOD and PPO, plants with W19 alone had significantly higher enzyme activity (**Figures 7**, **8**). In the general comparative performance of microbes with melatonin, BTL5 and melatonin application was a more synergistic treatment than W19 + melatonin. However, both inoculation of microbes and application of melatonin improved plant growth and performance under high salt concentrations.

**Figure 9:**
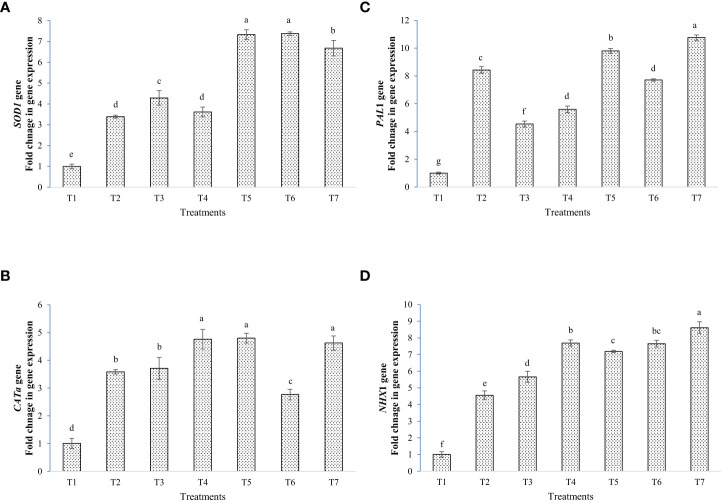
Fold changes in gene expression by the application of bacterial inoculants and melatonin, **(A)** *SOD*1, **(B)** *CAT*a, **(C)** *PAL*1, and **(D)** *NHX*1; T1=Negative control, T2=Positive control, T3 = 200 mM NaCl + Melatonin 20 ppm, T4 = 200 mM NaCl + *Bacillus safensis* BTL5, T5 = 200 mM NaCl + *Brevibacterium frigoritolerans* W19, T6 = 200 mM NaCl + *Bacillus safensis* BTL5 + Melatonin 20 ppm, and T7 = 200 mM NaCl + *Brevibacterium frigoritolerans* W19 + Melatonin 20 ppm. Error bars represent the standard deviation. The treatments with different letters are significantly different as determined by DMRT (p ≤ 0.05).

The results of gene expression studies showed that inoculation of bacterial strains and melatonin up-regulated salinity stress tolerance gene taken under study (**Figures 9A–D**). The expression of *SOD*1 gene was enhanced by seven folds in T5 and T6, as compared to the negative control. In *CATa* gene, expression was significantly enhanced by almost five times in T4, T5 and T7 against negative control. In T6, the expression was lower than the positive control. The *PAL*1 expression was highest in T7 followed by T5 against negative control. The expression was lower than positive control in T3, T4, and T6. In *NHX*1, eight times up-regulation was found in T7 plants followed by T4 and T6, as compared to the negative control.

**Figure 10:**
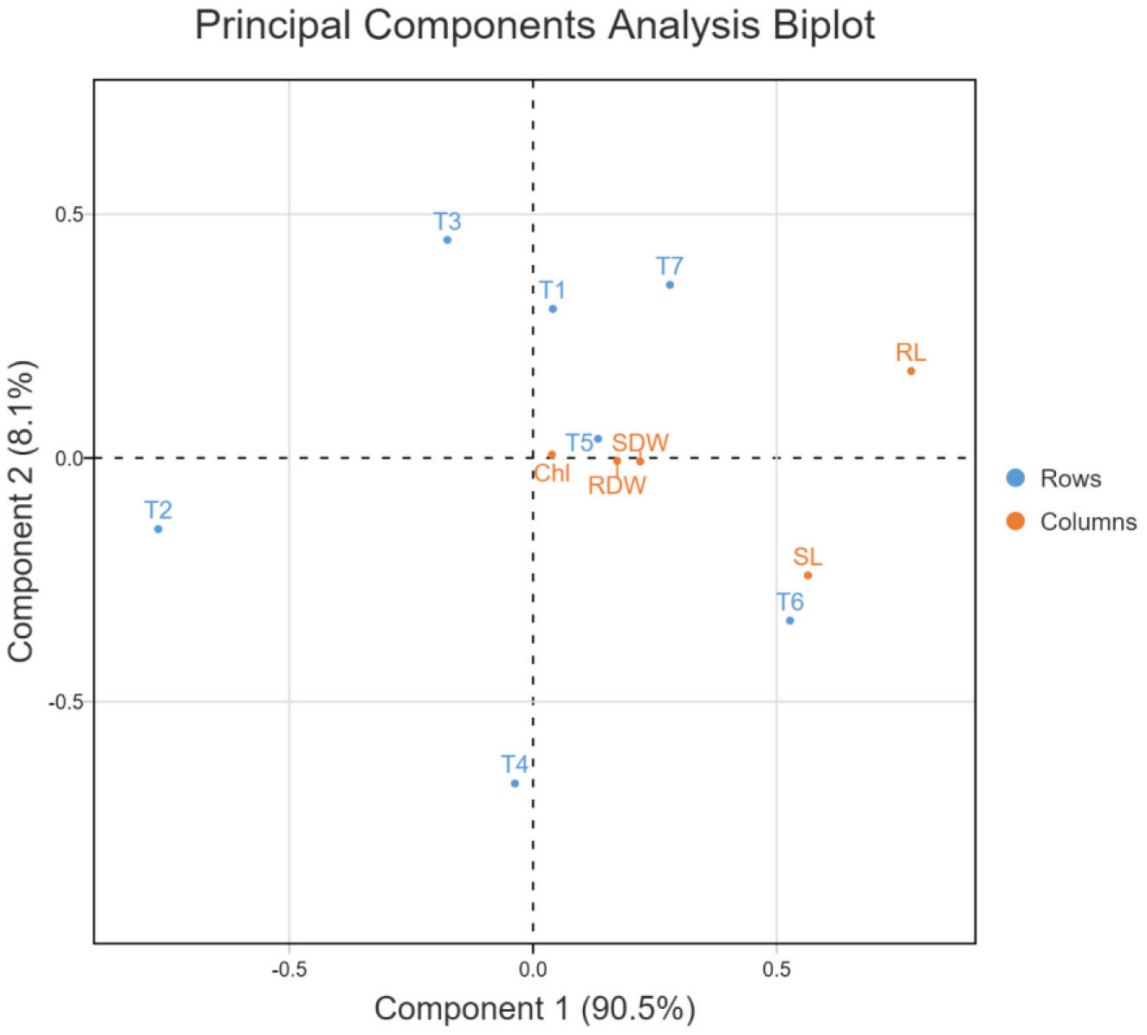
Principal component analysis biplot for growth attributes of rice plant as affected by bacterial inoculation and melatonin application under elevated salinity, Chl, Total chlorophyll; RL, Root length; SL, Shoot length; SDW, Shoot dry weight; RDW, Root dry weight.

**Figure 11:**
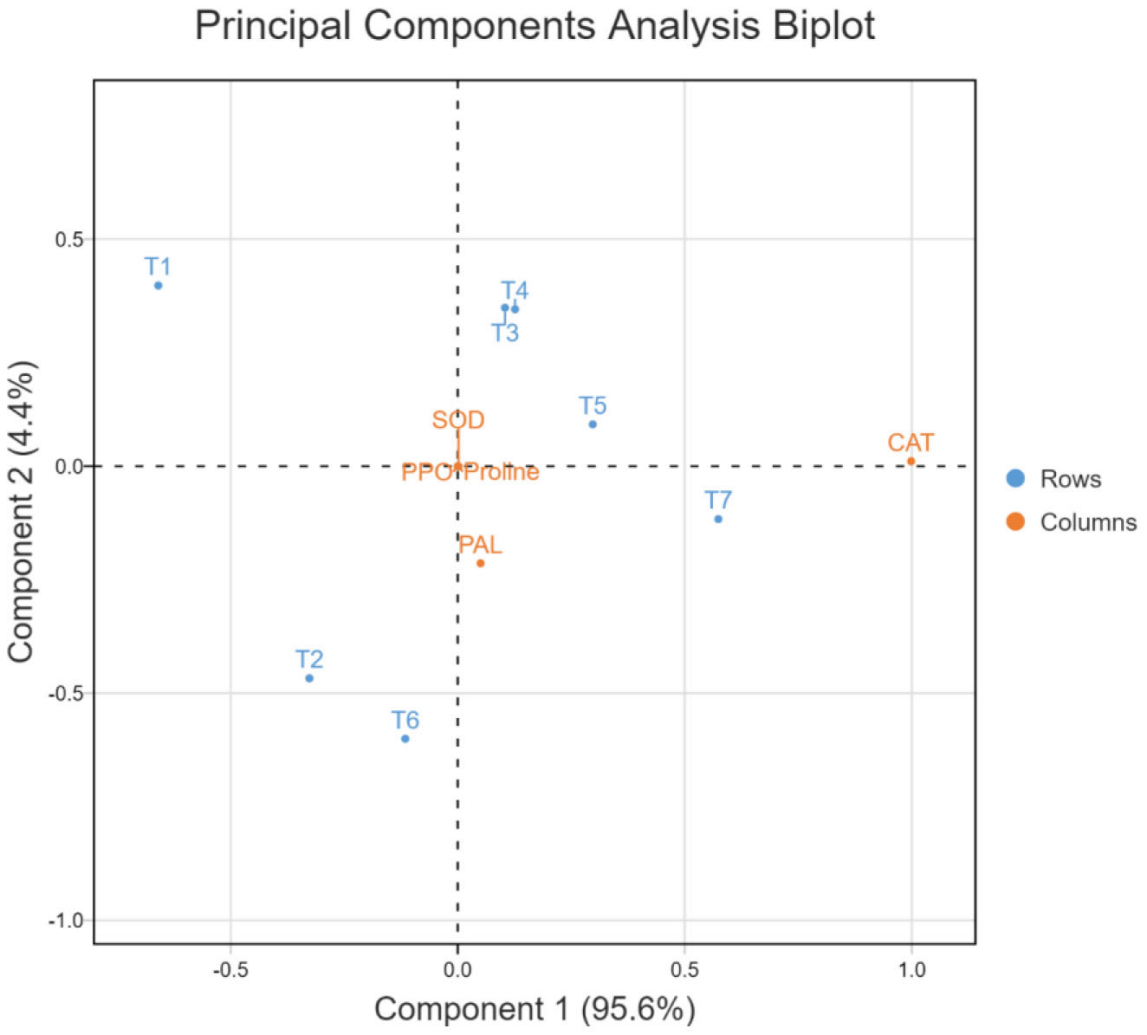
Principal component analysis biplot for ROS scavenging systems of rice plant as affected by bacterial inoculation and melatonin application under elevated salinity, SOD, Superoxide dismutase; CAT, Catalase; PPO, Polyphenol oxidase; PAL, Phenylalanine ammonia-lyase; PRL, Proline.

In the principal component analysis biplot of growth attributes, the first two principal components accounted for 98.6% of the variation (90.5 and 8.1%, respectively) in 7-dimensional space (**Figure 10**). In the PCA biplot for ROS scavenging systems, the first two principal components accounted for 100% of the variation (95.6 and 4.4%, respectively) in 7-dimensional space (**Figure 11**). In both PCA biplots, treatments with melatonin and bacterial inoculation were found to have a greater impact on growth and ROS scavenging under elevated salinity. In PCA of growth attributes, T7 (W19+Mel), T6 (2P2+Mel), and T5 (W19) were found to have higher impacts, similar to T1 (Negative control). This also indicates that albeit melatonin alone has effects on plant growth attributes, adding to the microbial inoculants resulting in a synergistic effect. In the case of PCA for ROS scavengers, the induced tolerance was higher for the bacterial inoculation alone, followed by an ensemble of bacteria plus melatonin.

## 4 Discussion

In the present study, bacterial strains were isolated, screened, and tested for their capability to support plant growth and development under salinity. Early seedling establishment, colonization efficacy, and synergism with the growth inducer compound melatonin were studied by two *in planta* trials.

The rhizosphere bacteria isolated in this study were found to have high salt tolerance (**Figure 2**), a fact that could be connected to their habitat. Saline soils harbor microbes that could survive under high salinity and perform plant growth-promoting activities under such conditions (Yadav and Saxena, 2018), and the crop rhizosphere is a rich habitat to search for trait-specific, salt-tolerant bacterial strains (Ling et al., 2022). High EPS production by these microbes could also contribute to salt tolerance (**Figure 3**) (Balsanelli et al., 2014; Singh et al., 2022), as EPS keeps a physical barrier between plant roots and the hypersaline environment (Singh and Jha, 2016; Sagar et al., 2022). These rhizospheric isolates were found to harbor multiple plant growth-promoting traits that can contribute to crop nutrition and productivity (**Figure 3**). The results are in line with the finding of Ling et al. (2022), which indicates that the rhizosphere is rich in microbes with specific functions. Microbes supplementing zinc and iron nutrition could help with nutrient fortification in grains (Singh et al., 2017) and help fight nutrient deficiency. The rhizospheric isolates were also found to have tryptophan-dependent as well as independent pathways for indole-3-acetic acid production (**Figure 3**). The presence of phytohormone-producing bacteria in the rice rhizosphere has been reported by earlier workers (Liu et al., 2021a). However, in the present study, bacteria with the tryptophan-dependent pathway were predominant.

The five isolates from this study belong to three genera: *Brevibacterium, Klebsiella*, and *Bacillus* (**Figure 1**). *Bacillus* is one of the very promising growth-promoting and salt stress alleviating bacterial agents reported in different host plants (Liu et al., 2021a; Sahu et al., 2021b). *Brevibacterium frigoritolerance* is reported as a salt stress alleviating bacteria from the wheat rhizosphere (Huang et al., 2022). Ibrahim and Ikhajiagbe (2021) reported *Klebsiella* and *Bacillus* as plant growth-promoting bacteria (PGPB) in rice. This kind of bacterial diversity is seen in the rhizospheres of different crops and has been linked to stress-relieving traits. It could also hold potential inoculants for saline soils.

Salinity reduced seedling emergence and growth, and inoculation of bacterial strains alleviated the effects of salinity (**Table 1**). This could be due to the reduction of Na^+^ toxicity by maintaining osmotic and ionic balance along with the reduction of oxidative stress. Endophytic and rhizospheric microbes are reported to improve germination and early seedling growth under salinity (Bai et al., 2017). These applied bacterial strains could be useful in a good plant stand at the nursery level.

Plant-microbe interactions are mediated by a complex cross-talk for successful colonization (Lahrmann et al., 2013). The present study showed good colonization of rhizospheric and endophytic bacteria in normal as well as under elevated salinity (**Figure 4**). The microbial association is decided by plant requirements (Gross, 2022). Our results evidence such principle, since the endophytic community was enriched with elevated salinity. A good amount of EPS production could also be one of the determinants contributing to the colonization competence of the rhizospheric and endophytic bacteria in the present study (Singh et al., 2022). EPS help bacteria adhesion to soil particles and plant roots while protecting microbes from the unfavorable environment. Good colonization could contribute to the stable *in planta* effects and is very much required for the popularization of microbial inoculants among the farming communities. Microbes with a wider host range could add to the utility of an inoculant for field application (Sahu et al., 2021b). Thus, microbes with high colonization competence to a wide range of crops at higher salinity can have high commercial value in the inoculant market. Colonization under elevated salinity could be an additional useful trait for the success of inoculation as it would further benefit the plants with added growth-promoting effects and increase nutrient uptake and tolerance under abiotic and biotic adversities. Jacquiod et al. (2022) recently hypothesized that continuous artificial shaping of microbiota in a plant can bring stabilization of microbial community structure that may be heritable to further generations. This gives experimental proof to the fact that inoculation of microbes with great plant growth-promoting and stress alleviating potential could substantially strengthen the plant function even for further generations.

In the pot trial, growth and biomass accumulation were improved by *Brevibacterium frigoritolerans* W19, *Bacillus safensis* BTL5, and melatonin (**Figure 5**). Since the overall plant function is a result of interaction between plants’ own genome and the plant-associated microbiome (Vandenkoornhuyse et al., 2015), the effects of the growth inducer molecule (melatonin) on the associated microbiome could be one of the crucial determinants in plant productivity under stress. Though some leads have been worked out in the case of AM fungi, where improvement in AM fungi colonization has been found due to exogenous melatonin application (Zhang et al., 2020), the area is still under-explored. Improvement in plant height and biomass accumulation was recorded from rice plants inoculated with bacteria and melatonin (**Figure 6**). These bacterial strains also produced IAA, which is reported by Kumar et al. (2021) to be a key inducer of seedling growth. The bacteria trigger salinity stress tolerance mechanisms of the plant (Sahu et al., 2021a). *Bacillus safensis* BTL5 had nutrient solubilizing and phytohormone-producing capability, which might have contributed to the salinity stress alleviation of rice plants and helped in higher nutrient uptake followed by improved utilization. The modulation of phytohormones and reduced osmotic/oxidative stress also contributed to the improved functioning of photosynthetic machinery (as evident from **Figure 7A**). Higher photosynthesis results in higher biomass accumulation, as seen in **Figure 6C, D**. Improvement in growth under salinity is crucially dependent on effective alleviation of oxidative and osmotic stress (Altaf et al., 2021). **Figure 8** shows the higher accumulation of enzymatic antioxidants from the inoculation of bacteria and melatonin. This could be due to induced systemic tolerance. Bacterial agents induce the over-expression of Na^+^/K^+^ antiporters to lock excess Na+ ions in the vacuoles along with the induction of aquaporins (Sahu et al., 2021b). This also maintains water homeostasis (Molina-Montenegro et al., 2020), which could be a reason for growth and development under high salinity.

In our previous study, *Bacillus safensis* BTL5 was reported to reduce salinity stress by increasing nutrient uptake, maintaining Na^+^/K^+^ balance, alleviating oxidative stress, and activating genes for osmotic adjustment (Sahu et al., 2021b). Together, all these effects would be responsible for the improved growth and development of rice. Induced systemic tolerance by inoculated bacteria and melatonin could be a key reason behind the induction of the ROS scavenging systems in rice (Vaishnav et al., 2020; Sahu et al., 2021b). In the present study, both proline-induced osmoregulation and maintenance of water homeostasis might play a role in maintaining the water status of salt-affected plants inoculated with *Bacillus safensis* BTL5+melatonin and *Brevibacterium frigoritolerans* W19 (Hayat et al., 2012; Shafi et al., 2019) (**Figures 7B, C**). Reduction in proline and other ROS scavenger’s content as observed in some bacterial and melatonin treatments is also a beneficial trait, which may possibly indicate that plants are experiencing lowered salt stress (Bhise et al., 2017).

The results of oxidative stress scavengers’ activity showed that melatonin improved certain parameters at a higher rate with one bacterial inoculant over the other, indicating preferential induction/synergism of some microbes with melatonin. Melatonin has been reported to regulate rhizogenesis and fortify reactive oxygen species (ROS) scavenging mechanisms, root architecture, photosynthetic capacity, and ionic balance of plants under salinity stress (Yan et al., 2020; Altaf et al., 2021).

The gene expression studies showed up-regulation of antioxidant genes by the application of melatonin with bacterial strains. Here, synergism was seen in the expression of *SOD*1, *CAT*a, and *PAL*1 expression. In addition, it indicated differential activation of salt tolerance genes by the inoculation of BTL5 and W19. Previous literature validated microbe-mediated induction of these genes for improving salinity mitigation in plants (Molina-Montenegro et al., 2020). The expression of *NHX*1 (Na^+^/H^+^ antiporter) gene was highest in all inoculated plants, and in the case of W19 + melatonin, the expression was further enhanced. This indicates the synergistic interaction of melatonin with W19. This upregulation in antiporter gene expression would be helping plants in the selective uptake of potassium and exclusion of excess sodium ions from plants’ system (Barragán et al., 2012). Up-regulation of the *NHX*1 gene by bacterial inoculation is reported earlier in our previous studies (Sahu et al., 2021b). The melatonin has also been earlier reported to up-regulate membrane transporters genes for salinity mitigation (Liu et al., 2020). In the case of *CAT*a gene, the BTL5 + melatonin treatment was having lesser expression than in BTL5 alone. This could be due to the activation of other catalase genes than *CAT*a, or the activation of other antioxidant machinery than *CAT*a. In the expression of *PAL*1, W19 and W19+melatonin was having higher expression than the positive control. Both BTL5 and W19 showed synergism with melatonin. Reduction in expression of some of the inoculated treatments than positive control is similar to the enzyme activity results, indicating reduced stress level in inoculated plants. *SOD*1 gene expression was high in W19 and BTL5 + melatonin inoculated plants as seen from enzyme activity studies where W19 got a higher accumulation of SOD. In gene expression, BTL5 showed synergism with melatonin. Such up-regulation of salt stress-responsive genes in rice by melatonin was earlier reported by Li et al. (2017).

Treatment with melatonin (T3) in rice was found to lead to higher plant growth and biomass compared with control plants under salinity. This increment in the growth and biomass accumulation could be related to the enhancement of the quantum efficiency of photosystem-II and thus the net photosynthesis rate by the addition of melatonin, as reported by Han et al. (2017) in rice. There have been a few reports on the improvement in plant performance after the combined application of microbes and melatonin (Liu et al., 2021b), which support our results that show synergistic effects with bacterial inoculants and melatonin. Yan et al. (2020) reported that pre-treatment with melatonin reduced membrane damage and oxidative stress in rice under salinity. Thus, the stage of application, as well as the concentration are crucial for the melatonin role. The present study reinforces the importance of melatonin in plants’ tolerance to salinity and establishes the compatibility of two bacterial inoculants with 20 ppm melatonin. Further studies are required to decipher the main determinants of synergism for the different bacterial inoculants.

The PCA biplots for growth attributes and ROS scavenging systems indicated the synergism between the bacterial inoculation and melatonin treatment (**Figures 9**, **11**). ROS scavenging is more impactful with the bacterial inoculation, which could be explained by the rhizospheric and endophytic bacterial roles in the reduction of the impact of abiotic stress by induction of systemic tolerance (Vaishnav et al., 2020; Sahu et al., 2021b). However, in the PCA of growth attributes, both melatonin and bacterial inoculation had higher impacts, which could be due to improvement in root system architecture and a reduction in sodium toxicity (Altaf et al., 2021; Verma et al., 2021).

## 5 Conclusion and future prospects

Exploring microbes with PGP traits and the ability of root colonization under high salinity may help in initiating symbiosis with the plant and protecting it against salinity stress. Also, strains having the ability of increased colonization under salinity stress could be a useful tool for successful field inoculation. Inoculation of *Brevibacterium frigoritolerans* W19 and *Bacillus safensis* BTL5 resulted in significant positive effects on rice, but there was a difference in colonization with the increase in salt concentration, *Bacillus safensis* BTL5 was found to have good colonization under elevated salinity. Though melatonin addition showed synergism with rhizospheric and endophytic bacterial inoculants, there was a difference in the induction of tolerance. Overall, exploring microbes with good colonization potential, stress alleviating attributes, and synergism with other growth-inducing compounds could be fundamental in making successful inoculants. The improved induction of plant tolerance with microbial inoculants and growth inducers should be further investigated.

## Data availability statement

The datasets presented in this study can be found in online repositories. The names of the repository/repositories and accession number(s) can be found in the article/**Supplementary Material**.

## Author contributions

RT, RS, PS conceived the idea and designed the experiments. AG performed the experiments. AG, PS and AS analyzed the data. RT, PS, AS and RS wrote and edited the manuscript. All authors contributed to the article and approved the submitted version.
