# Supplementary material for: Salinity alleviator bacteria in rice (Oryza sativa L.), their colonization efficacy, and synergism with melatonin
Source: Front Plant Sci. 2023 Jan 12;13:1060287. doi: 10.3389/fpls.2022.1060287 (PMC9878605; doi:10.3389/fpls.2022.1060287)
Supplement: Supplementary file 1 [file Table_1.docx]

| **S. No.** | **Genes** | **Oligo (5**'- 3'**)** |
| --- | --- | --- |
|  | Actin-F | TCCATCTTGGCATCTCTCAG |
|  | Actin-R | GTACCCTCATCAGGCATCTG |
|  | cCuZn-SOD1-F | GAGATTCCAAACCAGCAGGA |
|  | cCuZn-SOD1-R | TTGTAGTGTGGCCCAGTTGA |
|  | CATa-F | CCACCACAACAACCACTACG |
|  | CATa-R | CCAACGACTCATCACACTGG |
|  | PAL 1-F | CAGACACGGTCGTACCATTG |
|  | PAL 1-R | CCACCTCCTGCATTTGTTTT |
|  | *OsNHX1*-F | CGCGGCTGGGGGCTCTGTAC |
|  | *OsNHX1*-R | TCCCGACGGCTCCAAATAATGTG |

**Supplementary Table 1: RT-PCR primers used in the study**
